# Early Preventive Dental Visits: Do They Reduce Future Operative Treatments?

**DOI:** 10.3390/dj10040053

**Published:** 2022-03-31

**Authors:** Man Hung, Frank W. Licari, Martin S. Lipsky, Ryan Moffat, Val Joseph Cheever, Amir Mohajeri, Michael Stewart, Dean Orton, David Stewart

**Affiliations:** 1 College of Dental Medicine, Roseman University of Health Sciences, 10894 S. Riverfront Parkway, South Jordan, UT 84095, USA; flicari@roseman.edu (F.W.L.); mlipsky@roseman.edu (M.S.L.); rmoffat@roseman.edu (R.M.); jcheever@roseman.edu (V.J.C.); amohajeri@roseman.edu (A.M.); dorton@student.roseman.edu (D.O.); dstewart@roseman.edu (D.S.); 2 College of Engineering & Technology, Utah Valley University, 800 W. University Parkway, Orem, UT 84058, USA; michael.stewart98@hotmail.com

**Keywords:** dental operative procedures, prevention, pediatric, suburban, pediatric dentistry, oral health prevention

## Abstract

This study assessed the longitudinal impact of early preventive dental visits on the number of dental operative procedures in a prevention-oriented pediatric dental practice. Inclusion criteria consisted of patients zero to four years of age with at least two years of preventive services provided by the practice. Early preventive visits were the intervention and dental operative procedures were the assessed outcome. The goal was to determine if preventive visits at an early age decreased the number of operative procedures needed by the patient. The patients were divided into two groups: those with older siblings in the practice and those without older siblings in the practice. A secondary outcome was to compare these two patient groups to determine if a child who had older siblings previously treated in this preventive practice had better outcomes than those without siblings in the practice. ANCOVA tests were used to compare the average number of operative procedures in two age groups (<2 years and ≥2 years), and for those with and without dental insurance, in addition to children being younger sibling versus children without sibling, adjusting for the effect of covariates. The study sample consisted of 363 pediatric patients. Patients’ age at first visit ranged from 0 to 4 years old (mean = 2.13; SD = 1.15). The average number of operative procedures per year increased as the age at first visit increased (*p* < 0.05). The average number of operative procedures in two age groups (<2 years and ≥2 years) differed (*p* < 0.05) with those whose age at first visit ≥2 years experiencing more dental operative procedures than the younger group. The average number of operative procedures was similar between younger siblings (mean = 1.91; SD = 7.44) and children without siblings (mean = 1.54; SD = 2.1) (*p* > 0.05). The difference in the average number of operative procedures in children with insurance (mean = 1.59; SD = 5.25) and children without insurance (mean = 1.58; SD = 2.38) was non-significant (*p* > 0.05). More dental cleaning examinations were associated with fewer dental operative procedures (*p* < 0.05). These findings demonstrate that dental examinations before two years of age and more dental cleaning examinations lead to a decrease in the number of dental operative procedures needed by children.

## 1. Introduction

Despite being largely preventable, dental caries remains the most common chronic disease of children aged 6 to 11 years and adolescents aged 12 to 19 years [1]. In 2007, 28% of young children suffered from caries and 73% of these children required treatment [2]. Such a high caries incidence creates significant problems in these children, including inappropriate emergency room use, absenteeism from school, and caries-related pain [3].

Efforts by organized dentistry and others to address these concerns include emphasizing health promotion and disease prevention rather than a traditional disease-based treatment model. The American Academy of Pediatric Dentistry (AAPD) [4] recommends that every child have a dental home by one year of age. A core principle of this recommendation is that every child receives a caries risk assessment by the age of one in order to create a caries risk-based individualized preventive dental health program for the child. The goal of the one-year dental home guideline is to prevent dental disease before it ever occurs and to put into place a plan to keep dental disease from ever occurring. This guideline assumes that early intervention and prevention will decrease dental disease in children. Because a tooth is at risk to develop dental decay as soon as it erupts and a tooth erupting into a high caries risk oral cavity can be demineralized and decayed to the gingival level prior to being fully erupted, in high caries risk children, the only hope of trying to avoid high-cost restorative procedures early in their lives is to try to get a plan in place to prevent or at least decrease the severity of the dental decay that will occur if intervention does not take place. Thus, the best time to assess caries risk and create a prevention plan is before the teeth are decayed. Yet even today, only a small percentage of children visit the dentist by age 12 months as recommended by the AAPD [5]. A recent ADEA Policy Research Report reported the following, “As of November 2020, 37.5 million children were enrolled in Medicaid, and more than 6.5 million children were enrolled in the Children’s Health Insurance Program (CHIP). Although 43% of U.S. dentist participate in Medicaid or CHIP only about 20% of the children under age three enrolled in Medicaid get a dental visit for preventive services” [6]. Many of our highest risk children are not getting preventive dental visits early enough to prevent dental decay.

Despite the AADP’s recommendation of the concept of the one year first dental visit, research examining the benefit of early dental prevention visits on future treatment/cost has yielded mixed results. Several research studies found that children with early preventive dental care experienced better outcomes than children initiating later. In 2014, Nowak, Casamassimo, and Scott [3] confirmed the benefits of early preventive visits in high-risk Medicaid populations and reported that early starters subsequently needed less treatment for restorations, crowns, pulpotomies, and extractions than children with later dental intervention, despite having a similar caries risk. Treatment for early starters also costs $360 less over eight years of follow-up compared to late starters. Savage and colleagues [7], in 2004, studied North Carolina Medicaid enrollees from birth for 5 years and found that children whose age at first visit was less than one year were more likely to have subsequent preventive visits than children initiating care at age two to three years and that early preventive visits lowered dental costs. An Alabama study reported, “We found a positive impact of preventive dental visits on oral health. However, there is less evidence regarding the cost-effectiveness of preventive visits” among Children’s Health Insurance Program (CHIP) enrollees [8]. Another Alabama CHIP program study found no evidence that early preventive dental care led to fewer non-preventive visits or associated costs, but it did find that dental sealants, a procedure associated with preventive visits, did improve dental outcomes and lower costs [9].

Other research studies in the literature discussing early preventive dental visits seemed to be in opposition to the AAPD’s first year recommendation and failed to establish a relationship between early dental visits and less treatment use. Blackburn, Morrisey and Sen [10] found little evidence of the benefits of early preventive dental care in their retrospective Alabama Medicaid study and concluded that, “Preventive dental care from dentists appears to increase caries-related treatment, which is surprising. Additional research among other populations and beyond administrative data may be necessary to elucidate the true effects of early preventive dental care”. Using North Carolina Medicaid data, Beil and colleagues [11] compared caries-related treatment for children at age 43–72 months and found that children at highest risk benefited from a visit before age 18 months, but they suggested that children who did not have a high caries risk could delay their first dental visit until three years of age without an effect on subsequent dental outcomes if dental provider access was limited. Kranz et. al. [12] compared oral health outcomes of children receiving preventive oral health services within the ‘Into the Mouth of Babes’ program from primary care providers, dentists, or both, and they found that regardless of the provider type, children with early preventive dental care exhibited similar outcomes to children initiating dental care later, but that children that had only primary care provider visits “had a significantly greater proportion of DMFT [decayed, missing, and filled teeth], suggesting that more efforts are needed to improve referrals from primary care providers to dentists to help children obtain needed dental treatment”. A systematic review by Bhaskar, McGraw, and Divaris [13] examining the benefits of early preventive dental visits found limited evidence for supporting the effectiveness of early preventive dental care and that “more research among diverse populations is warranted”.

These conflicting results and the limited body of research in the literature examining the association between early preventive dental visits and subsequent dental operative procedures suggest a strong need for additional investigation. The purpose of this research study was to evaluate the effects of risk-based early preventive dental examinations on pediatric patients. The provider determined preventive dental recall examination frequency and preventive treatment based on a patient’s caries risk as determined at the initial new patient examination. Using data from a suburban, prevention-oriented, pediatric-dental-provider’s database, this study examined what impact, if any, early dental intervention measures have on long-term dental outcomes. The patient population included patients from zero to four years of age with at least two years of preventive services provided by the practice. Early preventive visits were the intervention and dental operative procedures were the assessed outcome. The goal was to determine if preventive visits at an early age decrease the amount of dental operative procedures needed by the patient. The patients were divided into two groups: those with older siblings in the practice and those without older siblings in the practice. This grouping allowed assessment of a secondary outcome comparing children with older siblings in the practice to those without siblings in the practice to determine if early preventive practices taught to parents regarding older siblings had an effect on the outcomes of the younger siblings.

This study sought to answer the following research questions:

1. What is the relationship between age at the first visit and the average number of dental operative procedures per year in children?
2. What is the relationship between a younger sibling’s average number of annual dental operative procedures and the difference between an older sibling’s age and younger sibling’s age of their initial visit?
3. What is the relationship between the total number of dental cleaning examinations and the total number of dental operative procedures?
4. Does the average number of annual dental operative procedures differ between children with insurance and children without insurance?
5. Does the average number of annual dental operative procedures differ between younger siblings and children without siblings?

## 2. Materials and Methods

### 2.1. Description of Data

This retrospective research study used data derived from a suburban, prevention-oriented, pediatric-dental-provider’s database and includes 600 patients seen from 2003 to 2015. Criteria for record extraction included patients receiving at least two years of dental preventive services rendered by the practice or patients seen in the practice who had other siblings receiving preventive services from the practice. Data elements included age at first visit, birth date, gender, dental cleaning examinations, dental operative procedures, and insurance types. Institutional Review Board exemption was obtained for this study by the Principal Investigator.

### 2.2. Practice Setting and Characteristics

The study used data extracted from the electronic dental records of a single pediatric dental provider, in solo practice, practicing in the same location throughout the study period in a suburban area near Salt Lake City, Utah. Practice reimbursement was mostly through non-government, third-party, fee-for-service traditional dental insurance (72%). Other payors included Medicaid (4%), self-pay (14%), and others (10%). The practice consistently provided caries prevention messaging, which was discussed at initial dental visits and typically reinforced at all follow-up visits.

The backbone of this prevention-focused pediatric dental practice was an assessment of periodontal and caries risk by history and examination, and the discussion of dental preventive techniques specific to the patient that could be utilized to decrease the child’s risk for decay and periodontal disease. These discussions included a demonstration of oral hygiene techniques specific to the child’s clinical presentation and age, the recommendation for an adult to brush and floss all children under the age of six in a supine position at least daily and preferably just before bedtime. The preventive messages also included a discussion of the caries demineralization process and how the frequency of eating or drinking fermentable carbohydrates and consuming processed food starches between meals can increase the demineralization process and potentially overpower the effect of twice-daily brushing. Other preventive recommendations included using fluoride toothpaste, regular preventive recall examinations, and fluoride varnish applications. Follow-up examination and ongoing discussion of these recommendations occurred at each dental visit.

### 2.3. Data Processing and Inclusion/Exclusion Criteria

Based on the Current Dental Terminology codes, patients with procedural codes for D (1510, 1515, 2150, 2160, 2330, 2331, 2332, 2391, 2392, 2393, 2930, 2933, 3220, 7140) were identified as having operative procedures and the procedural code for D (0120) as having a dental cleaning examination. Children with siblings seen in the dental practice were distinguished from children without siblings, and younger siblings were differentiated from older siblings based on their date of birth. Additionally, pediatric patients were grouped by age and insurance with those whose age at first visit was less than 2 years compared to those whose age of initial visit was equal to or greater than 2 years across insurance categories (with and without dental insurance).

For those with siblings treated in the dental practice, the analysis excluded older siblings with less than two years of dental preventive services from the practice and younger siblings who received only a single preventive service visit from the practice. Patients without siblings who had less than two years of preventive services rendered by the practice and those with a first visit after the age of 5 years were also excluded. Additional variables included the difference between an older sibling’s age of their initial visit and a younger sibling’s age of initial visit, the average number of dental operative procedures, and the average number of dental cleaning examinations.

### 2.4. Statistical Analyses

Descriptive statistics consisting of age of first visit, gender, number of dental operative procedures, and number of dental cleaning examinations were calculated. Pearson correlations between the age at the first visit, the number of dental cleaning examinations, and the number of dental operative procedures were reported. The study employed independent samples t-tests to compare the number of dental operative procedures between children without siblings in the practice versus the youngest sibling of those who had siblings treated in the same dental clinic practice. Additionally, the number of dental operative procedures across the difference between older sibling’s age of initial visit and younger sibling’s age of initial visit was analyzed. The study used ANCOVA to examine the group difference on the number of dental operative procedures controlling for covariates. We also conducted Levene’s test for homogeneity of variances across groups and applied Bonferroni correction for multiple comparisons. All statistical tests were conducted using SPSS 28 and the significance level was set at *p* < 0.05.

## 3. Results

The initial sample consisted of 600 pediatric patients whose age at first visit ranged from birth to 18 years old. After applying the inclusion and exclusion criteria, the final sample consisted of 363 patients. The age of the first visit ranged from 0 to 4 years old (mean = 2.13 years; SD = 1.15 years). The sample sizes for age at first visit were as follows: N = 17 children less than one year old, N = 111 for 1-year-old, N = 102 for 2-year-old, N = 75 for 3-year-old, and N = 58 for 4-year-old. Slightly under half of the children were female (48.5%), 64.7% were age 2 years or greater at first visit, 86% of children had dental insurance, and 79.6% were children with siblings also seen at the dental practice. The average number of annual dental operative procedures and the average number of annual dental cleaning examinations were 1.59 (SD = 4.95) and 1.51 (SD = 0.44), respectively. Table 1 displays the sample group characteristics.

Table 2 displays the relationship between the average number of dental operative procedures per year and age at the first visit in children. For children with siblings, the older they were at their first dental visit, the more dental operative procedures per year they had (r = 0.138; *p* < 0.05). This relationship was non-significant for children without siblings (r = 0.107; *p* > 0.05) (Table 2). Although there was an overall positive relationship between the age at the first visit and the average number of operative procedures, this relationship was not very strong. Table 2 also presents the relationship between the average number of dental cleaning examinations and the average number of operative procedures. For children with siblings seen in the dental practice, those who had more dental cleaning examinations tended to have significantly fewer dental operative procedures (r = −0.270, *p* < 0.05). Similarly, children with an initial visit at an age equal to or greater than two years experienced fewer dental operative procedures if they had more dental cleaning examinations (r = −0.327, *p* < 0.05) (Table 3). The average number of operative procedures in younger siblings (mean = 1.91; SD = 7.44) and in children without siblings (mean = 1.543; SD = 2.08) were similar (*p* > 0.05) (Table 4).

Levene’s test for homogeneity indicated that variances between various groups did not differ significantly (*p* > 0.05). There is a lack of a statistically significant difference between the younger sibling’s average number of operative procedures and the time between the older sibling’s age of initial visit and the younger sibling’s age of initial visit (r = 0.085, *p* > 0.05). Children who had their first dental visit when they were equal to or older than 2 years old tended to have more operative procedures on average than those who had their first dental visit when they were less than 2 years old (*p* < 0.05) (Table 4). Siblings and insurance status do not have statistically significant effect on the number of dental operative procedures (*p* > 0.15) (Table 4).

## 4. Discussion

Since the AAPD first introduced the recommendation for children to have a dental home by one year of age, little research has examined the impact of this recommendation among children whose dental home is in a suburban pediatric dental practice consisting of mostly non-government reimbursed dental insurance and fee-for-service patients. This study examined whether early dental visits within a suburban pediatric dental practice impacted children’s long-term dental outcomes. One important finding was that as the age at a first dental visit increases, the subsequent average number of dental operative procedures per year increases. This result supports the recommendation for early risk assessment and intervention advocated by dental professionals and their professional organizations. Since 1986, the AAPD [14] recommends that the first dental visit occur within six months of the eruption of the first tooth and no later than twelve months of age. However, previous research suggesting that early dental visits result in fewer overall treatments had limitations, such as drawing from multiple providers with variable treatment planning, small sample size, or pooling of data [7,8,15,16]. In addition, the data used in these prior studies did not ensure that early visits included education about preventive measures. The data used in this study were from a single dental provider who consistently provided caries risk-based, preventive visits, treatment, and education. The results of this study provide evidence that early intervention with consistent risk-based preventive messaging and follow-up can improve dental outcomes and supports the AAPD recommendations.

An important finding from this research study was that more dental preventive visits and dental cleaning examinations resulted in fewer dental operative procedures. This finding supports dental home recommendations for consistent risk-based examinations advocated by the AAPD [14]. Greater vigilance in adhering to a regular dental visit schedule can afford greater opportunity for more timely and consistent professional instruction and intervention, including increased opportunities for topical fluoride placement and enhanced adherence to home oral hygiene and eating recommendations. Seeing oral health professionals more regularly and connecting their child’s oral hygiene and dietary habits to their oral health may have also helped empower parents to be more involved in their young children’s oral health, which in turn may have led to the decreased need for operative surgical treatment of dental decay. A 2018 pilot study found that anticipatory guidance provided to mothers assists in reducing the prevalence of caries in children [17]. These early improvements in oral health may lead to long-term effects in overall health as oral health is a gateway to overall health and it can contribute to individuals’ general health and well-being [18].

There were two other notable findings in this study. The first was the influence of siblings upon the dental disease experience of the child. This study found no statistically significant difference between the average number of operative dental procedures between the younger sibling group and the children without sibling group. Whether or not a child had an older sibling previously treated in the practice did not affect the frequency of dental disease. The second was that there was no significant relationship between the younger sibling’s average number of operative dental procedures and the difference between the older sibling’s age of initial visit and the younger sibling’s age of initial visit. In this study, all children who were seen received subsequent early preventive dental visits based on their caries risk as determined by the AAPD’s guidelines on caries risk assessment. The fact that there was no difference between these groups speaks to the fact that caries risk is patient specific. If a high caries risk child is seen early and preventive interventions are put into place early, then dental operative treatments can be reduced. This study supports Beil and colleagues’ finding that high caries risk children benefit from early preventive dental visits [11]. It may also explain why researchers found that not all children may need the same number of early preventive dental visits [10,11]. If a caries risk assessment is not performed and subsequent early preventive dental visits are not based on caries risk, then large-scale retrospective studies not taking this into consideration can have variable results. The focus of patient care in this study was on the assessment of patient risk at the initial visit and the follow-up on the early preventive visits. Preventive treatments and follow-up exams were based on the patient’s caries risk in order to prevent the need for future dental operative procedures.

The results presented here should be considered in light of this study’s limitations and strengths. Limitations of this study include its relatively small sample size, a generally homogeneous pediatric patient population, and lack of information about their prior dental care. While other research studies correlating age at first visit and subsequent dental treatment used pooled data from Medicaid, with care by hundreds of providers with different training, background, and treatment planning philosophies; a strength of this study is the fact that it contains data from a single dental provider utilizing a simple, consistent, and preventive protocol over several years of longitudinal follow-ups. This consistent prevention messaging suggests that a dental provider making risk-based preventive messaging a routine part of practice can improve oral health outcomes as measured by future treatment frequency. A notable strength and weakness of this study is that most of the pediatric patients in this study were not receiving their dental services through government-funded dental insurance. While the described practice may be typical of other suburban practices, it may not be generalized to all providers, patients, and clinical settings. Future research to document that similar early dental risk-based prevention intervention can translate similar results to other settings and healthcare providers is needed. Future studies should also evaluate the degree and extent of caries prevention provided by focused, early, caries-risk based intervention appointments.

## 5. Conclusions

This study adds evidence to the literature that there is a relationship between children’s age at the first dental visit and the need for operative dental procedures. Children seen prior to age 2 for prevention-focused dental examinations resulted in fewer dental operative procedures than children seen for the first time after the age of two. In this study, more dental cleaning exams lead to a decrease in the number of dental operative procedures needed by children. By supporting the value of early intervention, regular dental care, and consistent messaging to caregivers, this study suggests that early and consistent dental prevention visits should continue to be a policy focus for the prevention of oral disease in children.

## Figures and Tables

**Table 1:** Demographic characteristics (N = 363).

Variables	Mean	SD	Median	Minimum	Maximum	N	%
Age at first visit (years)	2.13	1.15	2.00	0	4	363	100
<2 years old						128	35.3
≥2 years old						235	64.7
Number of operative procedures per year	1.59	4.95	0.62	0	87.16	363	100
Number of cleaning exams per year	1.51	0.44	1.65	0	2.41	363	100
Gender							
Male						187	51.5
Female						176	48.5
Insurance status							
No						51	14
Yes						312	86
Sibling status							
Children with sibling						289	79.6
Children without sibling						74	20.4

**Table 2:** Pearson correlation (r) between annual dental operative procedures and annual dental cleaning exams, age at first visit and children with and without siblings.

Avg. Number of Annual Dental Operative Procedures	N	r	*p*-Value
Overall sample			
Avg. number of dental cleaning exams per year	350	−0.250	<0.000
Age at first visit	363	0.131	0.013
Children with siblings			
Avg. number of dental cleaning exams per year	280	−0.270	<0.000
Age at first visit	289	0.138	0.019
Children without siblings			
Avg. number of dental cleaning exams per year	70	−0.209	0.083
Age at first visit	74	0.107	0.365

**Table 3:** Pearson correlation (r) between annual number of dental operative procedures and the annual number of dental cleaning exams across age groups.

Annual Number of Dental Operative Procedures with	N	r	*p*-Value
Annual number of dental cleaning exams			
when age at first visit < 2 years	128	−0.125	0.160
when age at first visit ≥ 2 years	235	−0.327	<0.001

**Table 4:** Difference in the number of operative procedures across various groups (multiple comparisons were adjusted using Bonferroni corrections).

Groups	Avg. Number of Operative Procedures (SD)	N	*p*-Value	Effect Size
Age at first visit			0.004 *	0.023
<2 years old	0.86 (1.44)	128		
>2 years old	1.98 (6.03)	235		
Sibling status			0.218 **	0.007
Children being younger siblings	1.91 (7.44)	144		
Children without siblings	1.54 (2.08)	74		
Insurance status			0.608 **	0.001
Children with insurance	1.59 (5.25)	312		
Children without insurance	1.58 (2.38)	51		

* Controlling for annual number of cleaning exam. ** Controlling for age at first visit and annual number of cleaning exam.

## Data Availability

The data presented in this study are available on request from the authors with Institutional Review Board approval.
